# Examining smartphone-assessed executive function metrics and intrinsic resting-state functional connectivity in depression

**DOI:** 10.1038/s41398-026-04300-2

**Published:** 2026-08-14

**Authors:** Julia M. Heckel, Aaron Clouse, David Klemballa, Joanna Hernandez, Jadyn Park, Alex D. Leow, Olusola A. Ajilore, Jessica A. Bernard, Sebastian Walther, Vijay A. Mittal, Stewart A. Shankman, Allison M. Letkiewicz

**Affiliations:** 1 Stephen M. Stahl Center for Psychiatric Neuroscience, Department of Psychiatry and Behavioral Sciences, Northwestern University Feinberg School of Medicine, Chicago, IL USA; 2 Department of Psychology, Northwestern University, Evanston, IL USA; 3 Department of Psychiatry, University of Illinois College of Medicine, Chicago, IL USA; 4 Department of Biomedical Engineering, University of Illinois at Chicago, Chicago, IL USA; 5 Department of Psychological and Brain Sciences, Texas A&M University, College Station, TX USA; 6 Texas A&M Institute for Neuroscience, Texas A&M University, College Station, TX USA; 7 Translational Research Center, University Hospital of Psychiatry and Psychotherapy, University of Bern, Bern, Switzerland; 8 Department of Psychiatry, Psychosomatics, and Psychotherapy, Center of Mental Health, University Hospital Würzburg, Würzburg, Germany; 9 Department of Psychiatry and Behavioral Health, The Ohio State University, Columbus, OH USA

**Keywords:** Human behaviour, Psychology

## Abstract

Major Depressive Disorder (MDD) is a common and costly mental health condition that is often associated with deficits in executive function. Smartphone applications (“apps”) have emerged as promising methods for assessing mental health-related outcomes in individuals’ daily lives that can detect changes that unfold over time. Although smartphone apps have been used to evaluate executive functioning in individuals with neurological conditions and in other mental health disorders, few studies have examined this in MDD. The present study tested whether smartphone-assessed executive function is (1) impaired in individuals with current MDD (cMDD; *n* = 30) relative to healthy controls (HC; *n* = 42) and (2) related to resting-state functional connectivity within cognitive control-related neural networks. For two weeks, participants completed a set-shifting (Trail Making Test, TMT-B) task on their smartphone. Participants with cMDD had significantly lower TMT accuracy (β = -0.24, *p* = 0.044) and greater variability in TMT accuracy (β = 0.25, *p* = 0.049) compared to HC. Resting-state analyses revealed that the association between TMT accuracy and connectivity between nodes of the dorsal attention network (bilateral intraparietal sulcus) was greater in HC than cMDD. The results extend previous laboratory-based findings by demonstrating that individuals with cMDD exhibit poorer mean-level smartphone assessed set-shifting performance and greater variability, along with altered set-shifting-related functional connectivity within the dorsal attention network. Smartphone-based assessments may offer a scalable and accessible approach for identifying executive function deficits in individuals with depression, which could be a useful tool for monitoring cognitive performance over time.

## Introduction

Major Depressive Disorder (MDD) is a highly prevalent mental health condition that affects approximately 17–20% of individuals over the course of their lifetime [1, 2]. Depression is also one of the leading causes of disability worldwide [3, 4]. Identifying markers of risk and course trajectory in depression is a critical endeavor, particularly with respect to potential mechanisms of depression, as this could reveal key processes that contribute to depression onset, persistence, recurrence, and response to treatment.

One potential marker of risk and course trajectory that is important to consider in MDD is executive function deficits, which are common in individuals with depression [5]; for review, see [6]. Executive functioning is an umbrella term that encompasses high-order cognitive processes, including set-shifting. Set-shifting, defined as the ability to flexibly switch (or “shift”) between different task sets or goals [7], has consistently been found to be disrupted in depression [8, 9]; for review, see [6]. Typically, set-shifting been assessed in the laboratory or clinic using standardized neuropsychological assessments, such as the Trail Making Test (TMT) and Wisconsin Card Sorting Task [8, 10]. Although in-person neuropsychological assessments have been instrumental in identifying executive function impairment in depression, these assessments have typically been used as one-time “snapshot” measures of executive function.

In contrast with a single assessment, repeated assessments of executive functioning can capture changes in executive function over time during naturalistic course or treatment [11]. Notably, repeated assessment methods, such as experience sampling methodology, have demonstrated day-to-day and within-day variability in executive function performance among adults with mild cognitive impairment [12, 13]. Variability may, in part, reflect dynamic changes in factors related to psychopathology, which cannot be captured by a one-time assessment. In particular, individuals with MDD may exhibit more variable cognitive performance as a result of clinically relevant fluctuations in state-dependent effects in mood, stress, rumination, and/or mind-wandering [14, 15]. While in-person repeated assessments in the clinic could reveal changes in executive functioning, such assessments would be impractical and burdensome. Limiting factors include the amount of time that would be needed to travel to the clinic, the need for evaluators to be available to repeatedly administer assessments, and potentially high evaluation costs. Increasingly, digital technologies, such as wearables and smartphone apps, have emerged as effective tools in the assessment and treatment of mental health disorders and related outcomes [16, 17]. Critically, these technologies offer several advantages over traditional in-person, lab or clinic-based assessments, including greater feasibility and scalability (e.g., remote assessment capacity, lower costs, and access to larger numbers of potential participants, including individuals from difficult-to-reach populations).

Smartphone-based assessments have frequently been used to evaluate executive function and changes in cognitive functioning in individuals with neurological disorders, such as in multiple sclerosis, frontotemporal lobe dementia, Alzheimer’s Disease, and Parkinson’s Disease; for review, see [18–21]. For example, smartphone-assessed executive function impairment has been identified in individuals with multiple sclerosis and linked to symptom onset and progression [22]. Digital assessments have also been used to assess executive function in a number of mental health disorders, including bipolar disorder, schizophrenia, and alcohol use disorder; for review, see [23–26]. By contrast, only a few studies have used smartphone-based executive function assessments in MDD. For example, one study with a non-clinical sample found that greater symptoms of depression were associated with poorer smartphone-based executive function [27]. However, additional studies are needed to clarify whether these effects extend to samples with clinical depression (MDD).

Executive functioning has robustly been linked with resting-state connectivity within several networks, including the frontoparietal network (FPN), and dorsal attention network (DAN), and salience network (SN) [28–30]. The FPN is central to cognitive control through its adaptation to changing task demands, primarily via the modulation and coordination of attention and working memory [31, 32]. The DAN supports top-down attentional mechanisms, particularly in the management of visuospatial attention [33]. The SN plays a crucial role in detecting and prioritizing relevant stimuli [34]. In healthy individuals, greater resting-state connectivity within the FPN, DAN, and SN is associated with better set-shifting performance [35–37]. Individuals with MDD often exhibit reduced resting-state connectivity within the FPN, DAN, and SN, which may reflect impairment in the ability to effectively engage executive function processes, such as set-shifting [29, 38–40]. However, no previous study has assessed whether resting-state connectivity relates to smartphone-based assessments of executive function in depression. Importantly, this could offer valuable insights into the validity of digital measures of executive function.

The primary goal of the present study was to examine whether smartphone-based assessments of set-shifting measured over two weeks are impaired in current MDD relative to healthy controls. The second aim was to examine whether resting-state functional connectivity in the FPN, DAN, and SN are related to the digital assessment measure, and whether patterns of connectivity differ between current MDD and healthy controls. Additionally, we sought to identify whether behavioral effects are dependent upon the duration of the assessment window – specifically, one day (i.e., one session), one week, or two weeks. As a follow-up, we tested whether variability (i.e., fluctuations/changes) in set-shifting differ by group. Given previous research indicating executive function deficits in set-shifting among individuals with current MDD (e.g.[6],), it was hypothesized that participants with current MDD would exhibit poorer and more variable performance on the set-shifting task than healthy controls. Additionally, it was hypothesized that smartphone-based executive function performance would be associated with resting-state functional connectivity within the FPN, DAN, and SN and that task-performance related connectivity would vary by group, such that task performance would be related to lower within-network connectivity in those with current MDD than healthy controls.

## Method

The present study includes a subset of participants from a larger transdiagnostic study of depression and psychomotor functioning (N = 305). Participants were recruited from clinics and the Chicago, IL community. Primary inclusion criteria for the larger study were: (1) 18–60 years of age, (2) able to read and comprehend English, (3) able to provide informed consent, (4) right-handed, and (5) met group-specific inclusion/exclusion criteria (described below). Primary exclusion criteria for the larger study were: (1) personal or first-degree family history of psychosis, mania, or hypomania, (2) head injury, (3) neurological conditions, tic disorders, or lifetime ADHD, (4) psychotropic medications that impact motor function (e.g., continuously administered medications that affect dopaminergic functioning, such as immunomodulators, anticonvulsants, and antidepressants such as bupropion and nortriptyline), (5) current moderate or severe alcohol/substance use disorder, and (6) a contraindication to the MRI scanning environment, such as a permanent metal (non-MRI safe) in the body, or current pregnancy. All procedures were approved by the Northwestern University Institutional Review Board. All participants provided written informed consent and were financially compensated.

For the present study, the primary inclusion criteria were (1) belonging to one of two groups: (a) healthy control (HC), defined as not meeting lifetime criteria for any major DSM-5 disorder, or (b) current MDD (cMDD); (2) completing at least one smartphone-based executive function assessment session over two weeks, and (3) completing a resting state MRI scan within 30 days of the smartphone assessment period. Group-specific inclusion/exclusion criteria were assessed using the Structured Clinical Interview for DSM-5 (SCID-5) [41].

### Measures and assessments

#### Structured clinical interview for DSM-5 disorders

Current and lifetime psychiatric diagnoses were assessed using the SCID-5 [41]. Diagnosticians were trained to criterion by watching SCID-101 training videos and completing three interviews observed by an advanced interviewer with diagnoses made in full agreement with the observer. Assessments were supervised by a licensed clinical psychologist (coauthor SAS) and regular supervision meetings were dedicated to discussing individual interviews and creating consensus ratings.

#### Smartphone application

Participants with an iPhone were asked to download BiAffect iOS software, which is freely available in the Apple App Store (https://www.biaffect.com) [42, 43]. Participants were provided with instructions for how to complete the smartphone version of the Trail Making Test (TMT-B) task and were instructed to complete it two times per week for two weeks. The BiAffect TMT was developed as a modified (digital) version of the TMT-B neuropsychological assessment, which is traditionally administered as a paper-and-pencil task [44, 45]. The digital TMT includes 13 circles that contain numbers 1 through 7 and letters A through F. Participants are told to connect the letters and numbers by alternating between tapping letters and numbers in order starting with the number 1 and ending with the number 7. The digital TMT task includes fewer circles than the standard TMT-B (which includes 25 circles) to accommodate smartphone screen size constraints [25]. Despite this difference, the digital TMT exhibits substantial convergent validity with the standard TMT-B (intraclass correlation = 0.74, 95% CI = 0.32–0.90) [25]. Participants’ task completion time and number of errors were recorded. TMT accuracy and TMT duration were significantly correlated, *r*(71) = 0.79, *p* = <0.001.

### Resting-State MRI

#### Data acquisition

Scanning was completed using a 3 tesla Siemens MAGNETOM Prisma scanner. The anatomical scan was collected using a high resolution T1-weighted magnetization-prepared rapid gradient-echo (MPRAGE) sequence (FOV = 256x256mm^2^, 1x1x1mm^3^ voxels, 178 slices, TR/TI/TE1, TE2, TE3/FA = 2170 ms/1190 ms/1.69 ms, 3.55 ms, 5.41 ms/7°). Resting state functional scans were collected using multiband accelerated EPI (FOV = 208x192mm^2^, 1x1x1mm^3^ voxels, 64 slices, TR/TE/FA = 555 ms/22 ms/47°, MB = 8). To ensure that enough high-quality resting state data was acquired, movement during the resting-state scan was monitored using Framewise Integrated Real-Time MRI Monitoring (FIRMM) software [46]. The resting state scan proceeded until at least 10 min of viable data were collected (framewise displacement [FD] ≤ 0.2 mm), with a maximum scan time of 16 min. Participants from the cMDD group had slightly longer total scan durations (total TRs: M = 1376.4, SD = 258.7) compared to healthy controls (total TRs: M = 1276.6, SD = 166.8), *t*(71) = 2.00, *p* = 0.049, as well as usable (FD ≤ 0.2 mm) TRs (cMDD: M = 1361.7, HC: M = 1266.0; *t*(71) = −2.03, *p* = 0.046). To account for potential effects of scan duration on results, participants’ total TRs and number of usable TRs were (separately) included as a covariate in follow-up analyses.

#### Anatomical and functional image preprocessing

The MPRAGE T1-weighted and BOLD EPI images were converted to Brain Imagine Data Structure (BIDS, v1.8.0) format using dcm2bids v3.1.1. Initial preprocessing steps were conducted for the anatomical and resting-state scans using fMRIPrep’s minimal preprocessing pipeline, version 20.2.0 (https://fmriprep.org) [47], a Nipype based tool (v1.5.1) [48, 49]. Each T1w (T1-weighted) volume was corrected for intensity non-uniformity using N4BiasFieldCorrection v2.1.0 [50] and skull-stripped using antsBrainExtraction.sh v2.1.0 (using the OASIS template). Brain surfaces were reconstructed using recon-all from FreeSurfer v6.0.1 [51], and the brain mask estimated previously was refined with a custom variation of the method to reconcile ANTs-derived and FreeSurfer-derived segmentations of the cortical gray-matter of Mindboggle [52]. Spatial normalization to the ICBM 152 Nonlinear Asymmetrical template version 2009c [53] was performed through nonlinear registration with the antsRegistration tool of ANTs v2.1.0 [54], using brain-extracted versions of both T1w volume and template. Brain tissue segmentation of cerebrospinal fluid, white-matter and gray-matter was performed on the brain-extracted T1w using fast (FSL v5.0.9) [55]. For full fMRIPrep anatomical preprocessing details, see the Supplement.

The BOLD EPI was skull-stripped and motion corrected (slice time correction was not done due to the MB accelerated acquisition). EPI was coregistered to a synthetic fieldmap and to the T1w structural image prior to registration to MNI 152 space. For full fMRIPrep functional preprocessing details, see the Supplement. Additional preprocessing steps were conducted using CONN toolbox (version 21a) [56, 57], which is a MATLAB-based toolbox. EPI data were smoothed using spatial convolution with a Gaussian kernel of 5 mm full width half maximum (FWHM). CONN’s standard “denoising” pipeline was implemented, which includes linear regression with the following noise variables: white matter (5 parameters), cerebrospinal fluid (5 parameters), motion (12 parameters), effect of rest (1 parameter), and scrubbing (person-specific), followed by temporal bandpass filtering. A 0.008–0.09 Hz band-pass filter was applied to remove high-frequency noise.

#### Image analysis

Region-of-interest (ROI) ROI-to-ROI correlation analyses were conducted within three resting-state networks – frontoparietal network (FPN), dorsal attention network (DAN), and salience network (SN). ROIs were based on predefined resting-state network parcellations that are built into CONN toolbox’s network atlas (32network, version 21a). CONN toolbox’s network atlas contains a total of 32 nodes across 8 networks, which were defined though an independent component analysis (ICA) that was implemented using a Human Connectome Project dataset with 497 subjects [57, 58]. The FPN, DAN, and SN networks included 15 ROI seeds, 4 of which are part of the FPN network: right lateral prefrontal cortex (FrontoParietal.LPFC r; predefined MNI coordinates [X, Y, Z]: [30, 38, 41]), left lateral prefrontal cortex (FrontoParietal.LPFC l; [−43, 33, 28]), right posterior parietal cortex (FrontoParietal.PPC r; [52, −52, 45]), left posterior parietal cortex (FrontoParietal.PPC l; [−46, −58, 49]); 4 of which are part of the DAN network: right intraparietal sulcus (DorsalAttention.IPS r; [39, −42, 54]), left intraparietal sulcus (DorsalAttention.IPS l; [−39, −43, 52]), right frontal eye fields (DorsalAttention.FEF r; [0, −6, 64]), left frontal eye fields DorsalAttention.FEF l; [−27, −9, 64]); and 7 of which are part of the SN network: anterior cingulate cortex (Salience.ACC; [0, 22, 35]), right anterior insula (Salience.Ainsula r; [47, 14, 0]), left anterior insula (Salience.Ainsula l; [−44, 13, 1]), right supramarginal gyrus (Salience.SMG r; [62, −35, 32]:), left supramarginal gyrus (Salience.SMG l; [−60, −39, 31]), right rostral prefrontal cortex (Salience.RPFC r; [27, 32, 46]), left rostral prefrontal cortex (Salience.RPFC l; [−32, 45, 27]).

### Data analytical approach

The final study sample included 72 participants (HC: *n* = 42; cMDD: *n* = 30) who (1) met group inclusion criteria, (2) had TMT assessment data available, and (3) had viable resting-state MRI scan data available. A series of linear regression models were conducted in R to assess whether individuals with depression exhibited poorer smartphone-assessed executive function task performance, which was measured over two weeks. For the TMT task, the effect of Group (dummy coded: HC = 0, cMDD=1) on accuracy (model 1a) and total completion time (model 1b). Primary analyses focused on mean-level outcomes, averaged across two weeks. Alternative time window analyses (one day and one week) focused on mean-level outcomes, averaged either across one day (i.e., only one time point was included) or one week. Exploratory analyses examined variability in outcomes, indexed as within-person standard deviation of the above variables over two weeks. Exploratory analyses also examined covariates, specifically IDAS subscales (anhedonia, general depression), medication (total medications, any medications), and scan duration (total TRs, usable TRs), which were included separately in models assessing TMT task performance and resting-state connectivity. Welch’s t-tests (two-sided, α = 0.05) were conducted for continuous demographic and clinical variables to assess differences in means between groups (e.g., age, total TRs, usable TRs). Chi-square tests were conducted for categorical demographic and clinical variables to assess differences in proportions between groups (e.g., race, medication status). Relevant test assumptions were checked and addressed as needed (e.g., Gaussian distributions, homogeneity of variance).

TMT accuracy and total duration were carried forward to the resting-state analyses, which were implemented using CONN Toolbox. Models separately tested whether Group (effect coded: HC = −1, cMDD=1) moderated ROI-to-ROI functional connectivity that was linked with the TMT outcomes. Analyses were limited to the eight resting-state (sub)networks of interest and were tested using cluster-level functional network connectivity multivariate parametric statistics, with false discovery rate (FDR) correction applied at the cluster level (corrected *p* < 0.05).

## Results

### Demographics and participant characteristics

As shown in Table 1, participants predominately identified as White, non-Hispanic, female sex, and female gender. On average, participants were 27.1 years old (*SD* = 8.7). The healthy control and cMDD groups did not significantly differ on age, education, ethnicity, or race. See Table 1 for additional demographics and participant characteristics. Across both groups, participants completed an average of 4.64 (SD = 2.99; range = 1–12) TMT task sessions, which did not significantly differ by group, *t*(67.07) = 1.50, *p* = 0.140.

**Table 1:** Demographics and Participant Characteristics.

Total (N = 72)		HC (n = 42)	cMDD (n = 30)	
		n (%)	n (%)	t-test/chi-square
Sex	Female	29 (69.0%)	24 (80.0%)	x²(1) = 1.08, *p* = 0.299
Gender	Female Male Something else (gender-fluid, non-binary, two-spirit, etc.)	28 (66.7%) 13 (31.0%) 1 (2.4%)	21 (70.0%) 6 (20.0%) 3 (10.0%)	x²(2) = 2.65, *p* = 0.266
				**-**
				**-**
Age (Years)	M (SD) Range (years)	26.7 (8.7) 18–52	27.7 (8.7) 19–57	t(70) = −0.50, *p* = 0.616
				**-**
Education	Some college or associate degree Bachelor’s degree Graduate or professional training	16 (38.1%) 13 (31.0%) 13 (31.0%)	11 (36.7%) 8 (26.7%) 11 (36.7%)	x²(2) = 0.29, *p* = 0.865
				**-**
				**-**
Ethnicity	Hispanic/Latine	9 (21.4%)	7 (23.3%)	x²(1) = 0.04, *p* = 0.848
Race	White Non-White Asian Black or African American More than one race/unknown	21 (50.0%) 21 (50.0%) 9 (21.4%) 4 (9.5%) 8 (19.0%)	16 (53.3%) 14 (46.7%) 5 (16.1%) 5 (16.1%) 4 (12.9%)	x²(1) = 0.08, *p* = 0.780
				**-**
				**-**
				**-**
				**-**
Medication	SSRI SNRI NDRI Benzodiazepine Other psychotropic	2 (4.8%) 0 (0%) 0 (0%) 0 (0%) 0 (0%)	4 (13.3%) 2 (6.7%) 4 (13.3%) 2 (6.7%) 1 (3.3%)	x²(4) = 9.64, *p* = 0.047
				**-**
				**-**
				**-**
				**-**
Number of Medications	0 1 2	40 (95.2%) 2 (4.7%) 0 (0%)	20 (66.7%) 7 (23.3%) 3 (10.0%)	x²(2) = 12.16, *p* = 0.002
				**-**
				**-**
IDAS-II Subscale Scores	M (SD) Well-being General depression	25.7 (6.8) 31.1 (8.2)	17.3 (6.2) 61.8 (10.7)	**-**
				t(70) = 5.44, *p* < 0.001
				t(68) = −13.53, *p* < 0.001

Values reflect original IDAS-II Well-being scores. For analyses, anhedonia was reverse-coded (Well-being multiplied by −1) and standardized (z-scored).

### TMT task performance

#### Set-shifting and depression: accuracy and task duration

**Fig. 1:**
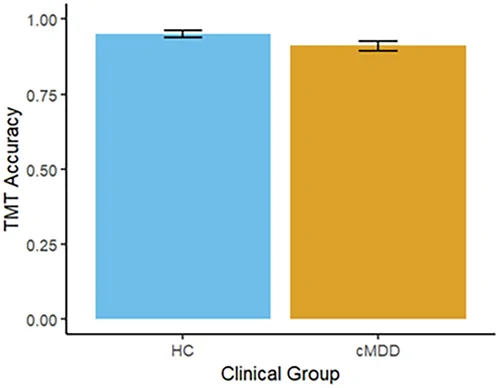
Group difference in mean-level Trail-Making Test (TMT) accuracy. Bar chart depicts the effect of Group on mean level TMT accuracy over two weeks. HC Healthy Controls, cMDD Current Major Depressive Disorder. Error bars represent ±1 standard error (SE).

There was a significant group difference for TMT accuracy, *t*(70) = −2.05, B = -0.04, β = −0.24, *p* = 0.044 across two-weeks of executive functioning assessment. As shown in Fig. 1, the cMDD group had lower accuracy than the HC group. There was a trend-level Group difference for TMT task duration, *t*(70) = 1.89, B = 3.25, β = 0.22, *p* = 0.063.

### Testing alternative (Shorter) assessment durations

#### Set-shifting and depression

##### 1 Day

There was not a significant group difference for TMT accuracy during the first session, *t*(70) = −0.60, B = -0.02, β = -0.07, *p* = 0.552. Similarly, there was not a significant group difference for TMT total task duration, *t*(70) = 0.35, B = 0.79, β = 0.04, *p* = 0.724.

##### 1 Week

There was not a significant group difference for TMT accuracy during the first week, *t*(70) = −1.52, B = −0.04, β = −0.19, *p* = 0.134. Similarly, there was not a significant group difference for TMT task duration from the first week only, *t*(70) = 2.96, B = 1.56, β = 0.18, *p* = 0.122.

### Resting-State MRI connectivity

#### Set-shifting, resting-state connectivity, and depression

##### TMT Accuracy

**Fig. 2:**
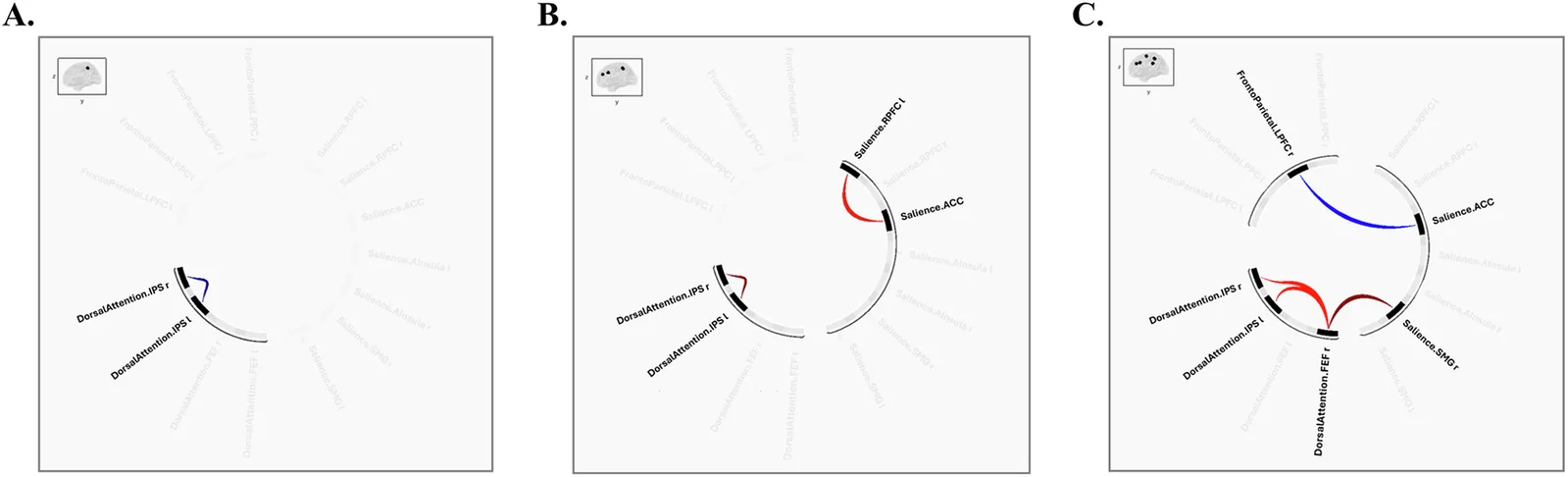
Resting-State Connectivity: Frontoparietal, Dorsal Attention, and Salience Networks. Network edges represent associations between resting-state connectivity and TMT accuracy. Red edges indicate increased connectivity, and blue edges indicate decreased connectivity. **A** TMT Accuracy x Group (Healthy Controls vs. cMDD). **B** Healthy Controls: TMT Accuracy. **C** Current MDD: TMT Accuracy.

No ROI-to-ROI main effects for TMT accuracy held following FDR correction (see the Supplement for uncorrected results). There was a significant interaction between Group and TMT accuracy on connectivity between the left and right DAN intraparietal sulcus, *F*(2,67) = 5.29, *p* = 0.044 (cluster corrected; see Fig. 2A). As shown in Fig. 2B, follow-up analyses identified that TMT accuracy was significantly related to greater left and right DAN intraparietal sulcus connectivity *t*(40) = 2.52, *p* = 0.016 in healthy controls only. Full connectivity results for the healthy control and cMDD groups are depicted in Figs. 2B, C and provided in Table 2. (Follow-up exploratory analyses that also included four Default Mode Network nodes were performed for TMT accuracy and task duration. For accuracy, the same DAN cluster result emerged with the DMN included. No other results held above FDR correction for accuracy or task duration.)

**Table 2:** Trail-Making Task (TMT) Accuracy: Healthy Control and cMDD Resting-State MRI Results.

Effect	Network	ROI 1	ROI 2	T Statistic (df)	p-unc
TMT Accuracy: HC	DAN	R IPS	L IPS	2.52 (40)	0.016
	SN	ACC	R RPFC	2.03 (40)	0.049
TMT Accuracy: cMDD	DAN-SN	R FEF	R SMG	3.20 (28)	0.003
	DAN	R IPS	R FEF	2.44 (28)	0.021
	DAN	L IPS	R FEF	2.40 (28)	0.023
	SN-FPN	ACC	R LPFC	2.12 (28)	0.043

##### TMT Task Duration

ROI-to-ROI main effects for TMT task duration held following FDR correction (see the Supplement for corrected results). There was no significant interaction between Group and TMT task duration.

### Exploratory analyses

#### Variability in accuracy and task duration

**Fig. 3:**
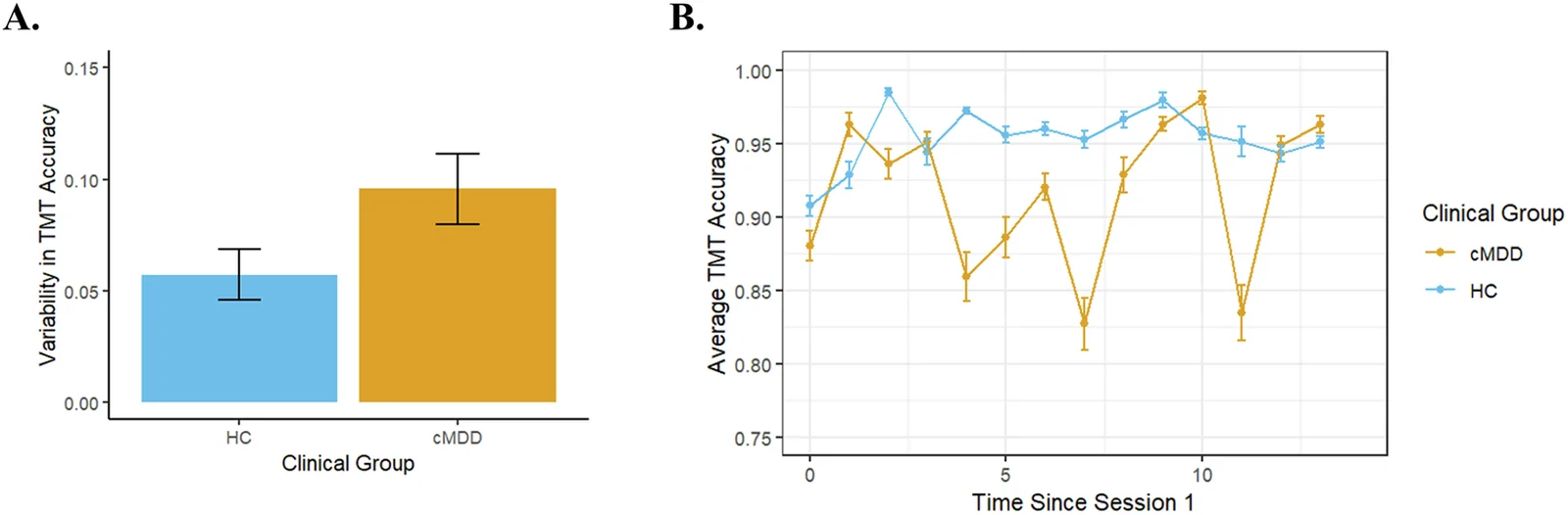
Group difference in variability in Trail-Making Test (TMT) accuracy. **A** Bar chart depicts the effect of Group on overall variability in TMT accuracy over two weeks. **B** Line graph depicts day-to-day fluctuations in average TMT accuracy across two weeks for both groups. HC Healthy Controls; cMDD Current Major Depressive Disorder. Error bars represent ±1 standard error (SE).

There was a significant group difference in variability of TMT accuracy (i.e., within-person standard deviation), *t*(59) = 2.01, B = 0.04, β = 0.25, *p* = 0.049 (note: 11 participants completed the TMT assessment at only one time point and, therefore, do not have variability scores). As shown in Fig. 3, variability in accuracy was greater in the cMDD than the HC group. By contrast, there was not a significant group difference in variability of TMT task duration, *t*(59) = 0.95, B = 1.01, β = 0.12, *p* = 0.345.

#### Time of day and TMT accuracy and task duration

Group differences in TMT accuracy and variability remained significant after including time of day that the TMT task was completed (based on a 24 h clock) as a covariate. Time of day also did not significantly predict TMT accuracy, variability, or task duration.

#### Covariate analyses

Including IDAS subscales (anhedonia, general depression) and medication (total medications, any medication) as covariates (separately) did not alter the behavioral or resting state connectivity results. Including TRs (usable TRs, total TRs) as covariates (separately) did not alter the resting state connectivity results. Additionally, none of the covariates significantly predicted TMT accuracy, variability, or task duration.

## Discussion

The present study identified that individuals with current MDD exhibited poorer smartphone assessed set-shifting (accuracy) than healthy controls over two weeks. Furthermore, resting-state connectivity analyses identified that the association between set-shifting accuracy and connectivity within the DAN varied by group. Specifically, the relationship between left and right intraparietal sulcus connectivity and set-shifting accuracy was stronger in healthy controls than in those with MDD. Follow-up analyses identified that individuals with MDD also had greater *variability* in their set-shifting accuracy over two weeks than healthy controls. This indicates that not only is set-shifting performance poorer in those with current MDD, but it fluctuates over time to a greater extent than in healthy controls. No significant group differences were observed in task duration (i.e., time to complete the task) – hence, the poorer accuracy in the MDD group was not likely the result of speeded responses (which can occur with a speed-accuracy tradeoff). In contrast with the standard paper-and-pencil TMT-B, which primarily indexes performance by completion time, the group differences in our study emerged for accuracy. Although the digital and standard TMT-B has shown evidence of strong convergent validity, the smartphone format may have contributed to our results (e.g., more touchscreen errors in the MDD group). Taken together, these results support the utility of using digital measures, which capture difficulties that individuals experience in the context of their daily lives, to assess executive functioning among individuals with depression.

These results align with prior research suggesting that executive function deficits are a core feature of major depressive disorder [5, 6, 8, 9] with set-shifting and cognitive flexibility being a particularly critical deficit [58, 59]. The results are also consistent with patient-reported experiences of day-to-day difficulties with cognitive functioning (e.g., attentional lapses, oscillating brain “fog”) and self-reported assessments of executive function, such as the Behavior Rating Inventory of Executive Functioning-Adult version (BRIEF-A) [60], which indicate that individuals with MDD experience more frequent and pronounced difficulties with executive function in daily life [61]. Critically, the present results extend prior research by demonstrating that impairments in set-shifting can be identified using a smartphone and without requiring a significant amount of time or burden – on average, individuals completed approximately 2–3 TMT tasks per week over the course of two weeks (4–5 total in total), each of which took participants less than a minute to complete.

Previous studies have shown that reduced activation in specific regions of the DAN, as well as reduced functional connectivity within the network, are associated with impairments in attentional control and cognitive flexibility [62]. Regarding the finding of altered connectivity between the left and right intraparietal sulcus specifically, the intraparietal sulcus has previously been found to support top-down attentional control, spatial working memory, prioritizing task-relevant stimuli, and maintaining spatial information [63]. Whereas increased resting-state DAN connectivity was linked with better set-shifting accuracy in healthy controls in the present study, this effect was not evident in the current MDD group. Set-shifting ability may depend, in part, on individuals’ capacity to utilize/bring bilateral attentional control and memory-related processes “online” to support task performance. Reduced resting-state DAN connectivity has previously been observed in individuals with MDD compared to healthy controls [29]. Additionally, prior studies have identified altered DAN connectivity in depression and associations with cognitive functioning [38, 64]. These results extend prior studies by linking DAN connectivity with smartphone-assessed set-shifting performance, which indexes individuals’ day-to-day cognitive performance and may more closely capture features of patient-reported experiences and difficulties in everyday life.

Although existing literature has implicated the DAN, FPN, and SN in cognitive control-related processes and stimuli detection [39, 65], no effects emerged within the FPN and SN networks. While prior studies have reported reduced within-network connectivity in the FPN and SN among individuals with MDD relative to healthy controls (for review, see [29, 40]), these studies have typically focused on group differences, not whether executive function is differentially associated with network connectivity. It is possible that the DAN or regions of the DAN (e.g., intraparietal sulcus) may be more central to set-shifting performance, at least set-shifting performance measured repeatedly over two weeks, than these other networks. More broadly, prior studies indicate that aberrant parietal cortex connectivity is common in MDD, such as hypoconnectivity between parietal regions of the DAN and the FPN (for review, see [29]. Functionally, altered parietal cortex connectivity is associated with impaired executive functioning and an increased tendency to engage in rumination/self-focused attention [66–68], as well as poorer clinical outcomes, including a reduced likelihood of remission following antidepressant treatment [69]. Previous studies have also linked altered parietal cortex connectivity to the course of MDD, including greater risk for depression onset and recurrence [70, 71]. Thus, our findings may not be set-shifting specific but could reflect a broader parietal cortex-related trait-like vulnerability for MDD. Prospective longitudinal studies are needed to determine whether the patterns identified in our study precede MDD onset and/or predict the clinical course of MDD.

Regarding the executive functioning assessment duration, no significant group differences were observed when examining only 1 TMT assessment or 1 week of TMT assessments. These findings suggest that longer assessment periods, such as two weeks, may be necessary to detect meaningful differences and fluctuations in set-shifting between groups [72]. It is also possible that requiring multiple within-day and more across-day assessments would have revealed group differences sooner than two weeks. It is important to note, however, that asking individuals to complete more assessments could result in greater participant burden and potentially less adherence/greater dropout over time.

There are several notable limitations of the present study. First, it will be important to assess other domains of executive function, such as updating working memory and fluency, in future studies. Only using one task for an executive function component (TMT for set-shifting) makes it difficult to determine if individuals have difficulties with a particular executive function or a specific task, the latter of which can result from difficulties with cognitive processes other than the primary construct of interest, such as working memory (sometimes referred to as the “task impurity problem”) [73]. A second limitation is that participants were only asked to complete the assessments twice per week for two weeks, which may have limited our ability to capture fluctuations in set-shifting over time. Third, practice effects across repeated assessments may have influenced task performance and variability, as participants became familiar with the task over time. As a result, performance improvement or stabilization due to practice effects may partially mask variability. Fourth, approximately one third of the MDD group reported taking psychotropic medication. While medication status was included to statistically covary for medication status, this approach does not fully eliminate (or control for) its potential impact on behavioral performance or resting-state connectivity. Fifth, our resting-state connectivity analyses were underpowered to detect small-sized interactions. Future studies with larger samples should be used to determine the replicability of our results, as well as identify other potential effects that may not have been detected. Sixth, resting-state connectivity was assessed at a single time point, making it unclear whether the observed associations reflect stable traits, temporary states, or dynamic fluctuations in network function. Finally, although not a limitation per se, the current study did not include self-report measures of executive function. Future studies could consider incorporating self-report measures (e.g., EMA items) alongside repeated smartphone-based assessments, as these approaches capture complementary aspects of cognitive functioning [74, 75].

The study also has several notable strengths. First, the study assessed an aspect of executive functioning, set-shifting, several times over two weeks, rather than relying on a single assessment. Second, the integration of the smartphone-based cognitive assessment with rs-fMRI revealed that an out-of-lab measure of set-shifting was related to altered DAN connectivity (specifically, bilateral intraparietal sulcus) in individuals with depression. Results also emphasize the potential utility of assessing executive function in depression using smartphone-based measures. Using smartphone assessments increases the feasibility and accessibility of assessing cognitive functioning in depression, particularly in the case of repeated assessments over time. Smartphone applications could help monitor clinically relevant changes in executive function, allowing clinicians to identify whether individuals are improving from interventions sooner. Notably, mindfulness and physical activity have emerged as promising behavioral interventions for targeting set-shifting and other executive functions [76–78]. More directly targeting and tracking improvement in executive functioning in combination with more traditional psychological and/or pharmacological interventions (CBT, antidepressant medication) could offer additional benefit for MDD treatment and the prevention of its recurrence (particularly among those with greater executive function impairments).

Overall, this study highlights the potential of smartphone-based assessments as useful tools for evaluating executive function impairment in depression. Targeting and tracking changes in executive function in conjunction with psychological and/or pharmacological interventions (CBT, antidepressant medication) could offer additional benefit for MDD treatment and the prevention of MDD recurrence, particularly among those with greater executive function impairment. Taken together, the integration of digital assessments of executive functioning along with targeted behavioral interventions offers opportunities for scalable, personalized approaches to treating depression. Future studies should aim to further validate smartphone-based measures of executive function in depression.

## Supplementary information

Examining Smartphone-assessed Executive Function Metrics and Intrinsic Resting-State Functional Connectivity in Depression Supplement

## Data Availability

Data supporting the findings of this study are hosted securely within the NIMH Data Archive (RDoCdb). Access is restricted to those who successfully submit a Data Use Certification request directly through the NIMH Data Archive portal under Collection ID: 2769, https://doi.org/10.15154/4an7-c536.

## Code availability

The code that supports the findings of this study is available from the corresponding author upon reasonable request.
