# Supplementary material for: Examining smartphone-assessed executive function metrics and intrinsic resting-state functional connectivity in depression
Source: Transl Psychiatry. 2026 Aug 14;16:628. doi: 10.1038/s41398-026-04300-2 (PMC13751617; doi:10.1038/s41398-026-04300-2)
Supplement: Supplementary file 1 [file 41398_2026_4300_MOESM1_ESM.docx]

Examining Smartphone-assessed Executive Function Metrics and Intrinsic Resting-State Functional Connectivity in Depression

**Supplement**

**Power Analysis**

Power analyses were implemented in R. The pwr package in R [1] was used to identify whether our study would be adequately powered to detect medium or medium-to-large sized main effects of group (which were based on meta-analytic evidence of effects this size for assessment-based measures of executive function in MDD). With a sample size of N=72, a medium or large-sized main effect could be detected with >90% power. We could also detect small-to-medium sized effects with >75% power. The InteractionPoweR package in R [2] indicated that there was sufficient power (>80%) to detect medium and large-sized interactions and approximately 75% power to detect small-to-medium-sized interactions, but analyses were underpowered to detect smaller interaction effects.

**fMRIPrep Preprocessing**

Results included in this manuscript come from preprocessing performed using *fMRIPrep* 20.2.0 [3, 4, RRID:SCR_016216], which is based on *Nipype* 1.5.1 [5, 6, RRID:SCR_002502].

**Anatomical data preprocessing**

A total of 1 T1-weighted (T1w) images were found within the input BIDS dataset.The T1-weighted (T1w) image was corrected for intensity non-uniformity (INU) with N4BiasFieldCorrection [7] distributed with ANTs 2.3.3 [8, RRID:SCR_004757], and used as T1w-reference throughout the workflow. The T1w-reference was then skull-stripped with a *Nipype* implementation of the antsBrainExtraction.sh workflow (from ANTs), using OASIS30ANTs as target template. Brain tissue segmentation of cerebrospinal fluid (CSF), white-matter (WM) and gray-matter (GM) was performed on the brain-extracted T1w using fast [FSL 5.0.9, RRID:SCR_002823, 9]. Brain surfaces were reconstructed using recon-all [FreeSurfer 6.0.1, RRID:SCR_001847, 10], and the brain mask estimated previously was refined with a custom variation of the method to reconcile ANTs-derived and FreeSurfer-derived segmentations of the cortical gray-matter of Mindboggle [RRID:SCR_002438, 11]. Volume-based spatial normalization to two standard spaces (MNI152NLin6Asym, MNI152NLin2009cAsym) was performed through nonlinear registration with antsRegistration (ANTs 2.3.3), using brain-extracted versions of both T1w reference and the T1w template. The following templates were selected for spatial normalization: *FSL’s MNI ICBM 152 non-linear 6th Generation Asymmetric Average Brain Stereotaxic Registration Model* [12, RRID:SCR_002823; TemplateFlow ID: MNI152NLin6Asym], *ICBM 152 Nonlinear Asymmetrical template version 2009c* [13, RRID:SCR_008796; TemplateFlow ID: MNI152NLin2009cAsym],

**Functional data preprocessing**

For each of the 1 BOLD runs found per subject (across all tasks and sessions), the following preprocessing was performed. First, a reference volume and its skull-stripped version were generated using a custom methodology of *fMRIPrep*. Susceptibility distortion correction (SDC) was omitted. The BOLD reference was then co-registered to the T1w reference using bbregister (FreeSurfer) which implements boundary-based registration [14]. Co-registration was configured with six degrees of freedom. Head-motion parameters with respect to the BOLD reference (transformation matrices, and six corresponding rotation and translation parameters) are estimated before any spatiotemporal filtering using mcflirt [FSL 5.0.9, 15]. The BOLD time-series (including slice-timing correction when applied) were resampled onto their original, native space by applying the transforms to correct for head-motion. These resampled BOLD time-series will be referred to as *preprocessed BOLD in original space*, or just *preprocessed BOLD*. The BOLD time-series were resampled into standard space, generating a *preprocessed BOLD run in MNI152NLin6Asym space*. First, a reference volume and its skull-stripped version were generated using a custom methodology of *fMRIPrep*. Several confounding time-series were calculated based on the *preprocessed BOLD*: framewise displacement (FD), DVARS and three region-wise global signals. FD was computed using two formulations following Power (absolute sum of relative motions) [16], and Jenkinson (relative root mean square displacement between affines) [15]. FD and DVARS are calculated for each functional run, both using their implementations in *Nipype* (following the definitions by [16]). The three global signals are extracted within the CSF, the WM, and the whole-brain masks. Additionally, a set of physiological regressors were extracted to allow for component-based noise correction [*CompCor*, 17]. Principal components are estimated after high-pass filtering the *preprocessed BOLD* time-series (using a discrete cosine filter with 128s cut-off) for the two *CompCor* variants: temporal (tCompCor) and anatomical (aCompCor). tCompCor components are then calculated from the top 2% variable voxels within the brain mask. For aCompCor, three probabilistic masks (CSF, WM and combined CSF+WM) are generated in anatomical space. The implementation differs from that of [16] in that instead of eroding the masks by 2 pixels on BOLD space, the aCompCor masks are subtracted a mask of pixels that likely contain a volume fraction of GM. This mask is obtained by dilating a GM mask extracted from the FreeSurfer’s *aseg* segmentation, and it ensures components are not extracted from voxels containing a minimal fraction of GM. Finally, these masks are resampled into BOLD space and binarized by thresholding at 0.99 (as in the original implementation). Components are also calculated separately within the WM and CSF masks. For each CompCor decomposition, the *k* components with the largest singular values are retained, such that the retained components’ time series are sufficient to explain 50 percent of variance across the nuisance mask (CSF, WM, combined, or temporal). The remaining components are dropped from consideration. The head-motion estimates calculated in the correction step were also placed within the corresponding confounds file. The confound time series derived from head motion estimates and global signals were expanded with the inclusion of temporal derivatives and quadratic terms for each [18]. Frames that exceeded a threshold of 0.2 mm FD or 1.5 standardised DVARS were annotated as motion outliers. All resamplings can be performed with *a single interpolation step* by composing all the pertinent transformations (i.e. head-motion transform matrices, susceptibility distortion correction when available, and co-registrations to anatomical and output spaces). Gridded (volumetric) resamplings were performed using antsApplyTransforms (ANTs), configured with Lanczos interpolation to minimize the smoothing effects of other kernels [19]. Non-gridded (surface) resamplings were performed using mri_vol2surf (FreeSurfer). Many internal operations of *fMRIPrep* use *Nilearn* 0.6.2 [20, RRID:SCR_001362], mostly within the functional processing workflow. For more details of the pipeline, see [the section corresponding to workflows in *fMRIPrep*’s documentation](https://fmriprep.readthedocs.io/en/latest/workflows.html).

**Copyright Waiver**

The above boilerplate text was automatically generated by fMRIPrep with the express intention that users should copy and paste this text into their manuscripts *unchanged*. It is released under the [CC0](https://creativecommons.org/publicdomain/zero/1.0/) license.

**Table S1. TMT Duration Results** (corrected and uncorrected)

| **Effect** | **Network** | **ROI 1** | **ROI 2** | **T Statistic (df)** | **p-unc** | **p-FDR** |
| --- | --- | --- | --- | --- | --- | --- |
| TMT Duration × Group | DAN | R IPS | L FEF | 2.24 (68) | 0.028 | 0.994 |
| TMT Accuracy Main Effect | DAN-SN | R FEF | L SMG | 2.42 (68) | 0.018 | 0.711 |
|  | DAN-SN | R FEF | R SMG | 2.24 (68) | 0.028 | 0.711 |
|  | DAN-SN | L IPS | L SMG | 2.19 (68) | 0.032 | 0.711 |
|  | DAN | L IPS | R FEF | 2.11 (68) | 0.038 | 0.711 |
|  | DAN-FPN | L IPS | R LPFC | 2.07 (68) | 0.042 | 0.711 |
|  | DAN-SN | R FEF | L RPFC | 2.02 (68) | 0.047 | 0.711 |
| TMT Duration Main Effect | DAN-SN | R IPS | L SMG | -3.07 (68) | 0.003 | 0.246 |
|  | DAN-SN | L IPS | L SMG | -2.83 (68) | 0.006 | 0.246 |
|  | DAN-FPN | R FEF | R LPFC | -2.59 (68) | 0.012 | 0.246 |
|  | DAN-SN | R FEF | L SMG | -2.54 (68) | 0.013 | 0.246 |
|  | DAN-FPN | R FEF | L LPFC | -2.52 (68) | 0.014 | 0.246 |
|  | DAN-FPN | R IPS | R LPFC | -2.52 (68) | 0.014 | 0.246 |
|  | SN-FPN | ACC | L PPC | 2.44 (68) | 0.017 | 0.246 |
|  | DAN-SN | R FEF | L RPFC | -2.41 (68) | 0.019 | 0.246 |
|  | DAN-SN | L FEF | L SMG | -2.34 (68) | 0.022 | 0.259 |
|  | DAN-FPN | R IPS | L LPFC | -2.14 (68) | 0.036 | 0.340 |
|  | DAN-SN | R FEF | R SMG | -2.12 (68) | 0.038 | 0.340 |
|  | DAN | R IPS | R FEF | -2.11 (68) | 0.039 | 0.340 |
|  | FPN | R PPC | L LPFC | 2.02 (68) | 0.047 | 0.376 |

**Figure S1.**

**A. TMT Accuracy Main Effect** (uncorrected results)

**Figure S2.**

**A. TMT Task Duration (RT) Main Effect** (uncorrected results)

**B. TMT Duration x Group (Healthy Controls vs. cMDD) Effect** (uncorrected results)

**References**

1. Baranger DAA, Finsaas MC, Goldstein BL, Vize CE, Lynam DR, Olino TM. Tutorial: power analyses for interaction effects in cross-sectional regressions. *Adv Methods Pract Psychol Sci* 2023; **6**: 25152459231187531.
2. Champely S. pwr: Basic Functions for Power Analysis. R package version 1.3-0; 2023. Available at: <https://github.com/heliosdrm/pwr>.
3. Esteban O, Markiewicz CJ, Blair RW, Moodie CA, Isik AI, Erramuzpe A *et al.* fMRIPrep: a robust preprocessing pipeline for functional MRI. *Nat Methods* 2019; **16**: 111–116.
4. Markiewicz CJ, Esteban O, Goncalves M, Poldrack RA, Gorgolewski KJ. fMRIPrep: a robust preprocessing pipeline for functional MRI. 2026. doi:[10.5281/ZENODO.852659](https://doi.org/10.5281/ZENODO.852659).
5. Gorgolewski K, Burns CD, Madison C, Clark D, Halchenko YO, Waskom ML *et al.* Nipype: a flexible, lightweight and extensible neuroimaging data processing framework in Python. *Front Neuroinform* 2011; **5**. doi:[10.3389/fninf.2011.00013](https://doi.org/10.3389/fninf.2011.00013).
6. Gorgolewski KJ, Esteban O, Ellis DG, Notter MP, Ziegler E, Johnson H et al. Nipype: a flexible, lightweight and extensible neuroimaging data processing framework in Python. 0.13.1. 2017. doi:10.5281/ZENODO.581704.
7. Tustison NJ, Avants BB, Cook PA, Yuanjie Zheng, Egan A, Yushkevich PA *et al.* N4ITK: improved N3 bias correction. *IEEE Trans Med Imaging* 2010; **29**: 1310–1320.
8. Avants B, Epstein C, Grossman M, Gee J. Symmetric diffeomorphic image registration with cross-correlation: evaluating automated labeling of elderly and neurodegenerative brain. *Med Image Anal* 2008; **12**: 26–41.
9. Zhang Y, Brady M, Smith S. Segmentation of brain MR images through a hidden Markov random field model and the expectation-maximization algorithm. *IEEE Trans Med Imaging* 2001; **20**: 45–57.
10. Dale AM, Fischl B, Sereno MI. Cortical surface-based analysis. *Neuroimage* 1999; **9**: 179–194.
11. Klein A, Ghosh SS, Bao FS, Giard J, Häme Y, Stavsky E *et al.* Mindboggling morphometry of human brains. *PLoS Comput Biol* 2017; **13**: e1005350.
12. Evans AC, Janke AL, Collins DL, Baillet S. Brain templates and atlases. *Neuroimage* 2012; **62**: 911–922.
13. Fonov V, Evans A, McKinstry R, Almli C, Collins D. Unbiased nonlinear average age-appropriate brain templates from birth to adulthood. *Neuroimage* 2009; **47**: S102.
14. Greve DN, Fischl B. Accurate and robust brain image alignment using boundary-based registration. *Neuroimage* 2009; **48**: 63–72.
15. Jenkinson M, Bannister P, Brady M, Smith S. Improved optimization for the robust and accurate linear registration and motion correction of brain images. *Neuroimage* 2002; **17**: 825–841.
16. Power JD, Mitra A, Laumann TO, Snyder AZ, Schlaggar BL, Petersen SE. Methods to detect, characterize, and remove motion artifact in resting state fMRI. *Neuroimage* 2014; **84**: 320–341.
17. Behzadi Y, Restom K, Liau J, Liu TT. A component based noise correction method (CompCor) for BOLD and perfusion based fMRI. *Neuroimage* 2007; **37**: 90–101.
18. Satterthwaite TD, Elliott MA, Gerraty RT, Ruparel K, Loughead J, Calkins ME *et al.* An improved framework for confound regression and filtering for control of motion artifact in the preprocessing of resting-state functional connectivity data. *Neuroimage* 2013; **64**: 240–256.
19. Lanczos C. Evaluation of noisy data. *J Soc Ind Appl Math Ser B Numer Anal* 1964; 1: 76–85.
20. Abraham A, Pedregosa F, Eickenberg M, Gervais P, Mueller A, Kossaifi J *et al.* Machine learning for neuroimaging with scikit-learn. *Front Neuroinform* 2014; **8**. doi:[10.3389/fninf.2014.00014](https://doi.org/10.3389/fninf.2014.00014).
